# Progress in Medicine

**Published:** 1902-11-01

**Authors:** 


### PROGRESS IN MEDICINE.

(*Continued from page* 67.)

**Blackwater Fever.**—A remedy for blackwater fever used with success by the natives of East Africa is introduced to notice by D. R. O'Sullivan Beare.[59] It consists of a watery decoction of the root of a new species of cassia—christened *Cassia beareana* by Mr. Holmes—and has yielded excellent results in Dr. Beare's hands. The latter expresses his view that blackwater fever is a toxæmia, strongly predisposed to by the cachexia, but not otherwise connected with malaria, and not due to cinchonism (He has observed it in natives who have never tasted quinine). It is not benefited by quinine. The toxin destroys the red blood corpuscles, and the hæmoglobin thus set free accounts for the various symptoms—fever, vomiting, jaundice, hæmaturia. Its passage through the kidneys sets up a true nephritis from which frequently result suppression of urine and uræmia. The new remedy seems equally beneficial in bilious remittent fever. C. F. Harford-Battersby,[60] whilst cordially welcoming the new drug, dissents from these views as to the etiology and pathology of blackwater fever. He maintains the disease is a manifestation of malaria, and is benefited by quinine, and suggests that since *Cassia beareana* seems peculiarly useful in the vomiting of this disease, it should be tried for other cases of vomiting. No benefit resulted from its use in a case of blackwater fever under Dr. F. W. Smith recently treated in the London Hospital.[61] The case, which terminated fatally, was marked by persistent vomiting. Much less fluid was, however, given than is used by the natives. The only naked eye change *post mortem* was deep pigmentation of the organs, especially of the kidneys. The patient had had malaria frequently, and some parasites were found.

**Malaria.**—Dr. Manson[62] treats of the diagnosis of malaria in England from three standpoints: (*a*) the clinical; (*b*) the therapeutical; (*c*) the microscopical. (*a*) The clinical diagnosis.—Fever, anæmia, icturus, enlarged spleen and liver are present in many other conditions. Periodicity, recurrence of symptoms every 48 or 72 hours, is the most important point in diagnosis. *Quotidian* periodicity can be quite disregarded as a basis for diagnosing malaria. True quotidian periodicity rarely if ever occurs in the disease. Quotidian fever associated with enlarged spleen and anæmia or enlarged liver is pretty certainly due to something else, not to malaria. (*b*) The therapeutical diagnosis.—Quinine is invaluable here. A periodic fever which resists quinine given and absorbed in full doses for three or four days is not malaria. Attention must be paid to absorption. Pills and tabloids are useless; sometimes hypodermic injections of the acid hydrochloride must be used. (c) The microscopical diagnosis.—This, *in the hands of one accustomed to the work*, is invaluable if the patient is not under the influence of quinine at the time, although a marked leucocytosis of the large mononuclear form is, even then, suggestive. Under quinine the parasites rapidly disappear from the blood. To make the blood films cleanse with alcohol the finger-tip and a microscope slip, prick the finger and by contact, get a minute drop of blood on to the slip, then roll it out with the shaft of a long sewing needle. The hypodermic injection of solutions of quinine finds favour in India.[63] The bi-hydrochloride gives the least pain. The injections should not be too superficial. A. Powell gives as much as 15 or even 18 grains in one injection and has never had any untoward results in a large series of cases. G. B. Ferguson[64] strongly advises the bi-hydrobromate. Six injections of 3 grains each, one every alternate day, suffice. P. Kuhn,[65] of South West Africa, considers malaria and horse-sickness allied, and claims to have observed great benefit by inoculations with the curative serum used for horse-sickness. M. Armand Gautier[66] describes how he was first led to use cacodylates in the treatment of malarial cachexia. He finds that *nouveau cacodylate* or *arrhénal*, bisodium methyl arsenate, is a non-toxic preparation of arsenic very useful in malaria. He injects 5 centigrams hypodermically. The Italian Society for the Study of Malaria[67] finds that the more soluble salts of quinine, the bisulphate, hydrochlorate, and ethyl carbonate (euchinin) are valuable prophylactics, more so than the compounds of ircn, arsenic and quinine, which under the name of "esanophele" are advocated by Professor Grassi,[68] and approved by Professor Guiart. Ray Lankester[69] suggests a convenient terminology for the various stages of the malarial parasite. The nomenclature aims at simplicity and treats the parasite from the point of view of malaria. In Barbadoes[70] no indigenous malaria exists, and careful search has failed to find any *anopheles*. Filaria is, however, very rife, and *culex fatigans*, which acts as an efficient host for this disease, abounds.

[59] Lancet, Feb. 1. [60] Ibid., March 29, p. 920. [61] Clin. Jour., April 16, p. 411. [62] Ibid., June 4. [63] Brit. Med. Jour., May 3. [64] Ibid., Feb. 22. [65] Ibid., Epitome, July 4. [66] Les Nouveaux Remèdis, Feb. [67] Brit. Med. Jour., April 5, p. 859. [68] Lancet, July 5, p. 37. [69] Brit. Med. Jour., March 15. [70] Ibid., June 14.

